# Enhanced Determination of *Streptococcus pneumoniae* Serotypes Associated with Invasive Disease in Laos by Using a Real-Time Polymerase Chain Reaction Serotyping Assay with Cerebrospinal Fluid

**DOI:** 10.4269/ajtmh.2010.10-0225

**Published:** 2010-09

**Authors:** Catrin E. Moore, Amphone Sengduangphachanh, Thaksinaporn Thaojaikong, Joy Sirisouk, Dona Foster, Rattanaphone Phetsouvanh, Lesley McGee, Derrick W. Crook, Paul N. Newton, Sharon J. Peacock

**Affiliations:** Wellcome Trust-Mahosot Hospital-Oxford Tropical Medicine Research Collaboration, Mahosot Hospital, Vientiane, Laos; Centre for Clinical Vaccinology and Tropical Medicine, Nuffield Department of Clinical Medicine, Churchill Hospital, University of Oxford, Oxford, United Kingdom; Nuffield Department of Medicine, University of Oxford, John Radcliffe Hospital, Headington, Oxford, United Kingdom; Respiratory Diseases Branch, Division Bacterial Diseases, National Center for Immunization and Respiratory Diseases, Centers for Disease Control and Prevention, Atlanta, Georgia; Department of Microbiology and Immunology, Faculty of Tropical Medicine, Mahidol University, Bangkok, Thailand; Department of Medicine, University of Cambridge, Addenbrooke's Hospital, Cambridge, United Kingdom

## Abstract

A prospective hospital-based study was undertaken to define the incidence of invasive pneumococcal disease (IPD) and circulating serotypes in Laos. Of 10,799 patients with hemocultures and 353 patients with cerebrospinal fluid samples, 0.21% and 5.4%, respectively, were positive for *Streptococcus pneumoniae*, giving a total of 35 IPD patients. We developed a real-time polymerase chain reaction to detect serotypes represented in the 13-valent pneumococcal vaccine. A blinded evaluation comparing serotype as defined by the Quellung reaction versus the polymerase chain reaction demonstrated 100% concordance. The most frequent serotype (n = 33 patients) was 1 (n = 6), followed by serotypes 5, 6A/B/C, 14, and 23F. Serotypes represented in the 7-valent polysaccharide-protein conjugate vaccine (PCV-7) infected 39% of patients, with 73% coverage for the PCV-10 and PCV-13 vaccines. Although the sample size is small, these data suggest that the PCV-7 vaccine may have relatively low efficacy in Laos. Further studies are urgently needed to guide pneumococcal vaccine policy in Laos.

## Introduction

Invasive pneumococcal disease (IPD) is a major cause of morbidity and mortality with an estimated 1.6 million persons dying of pneumococcal disease each year, of which 0.7–1 million are children less than five years of age living in the developing world.^1^ The introduction of a seven-valent polysaccharide-protein conjugate vaccine (PCV-7, Prevnar™; Wyeth Lederle Vaccines, Pfizer, New York, NY) has led to a reduction in the rate of IPD in the United States and other developed countries.^2^,^3^ Less data on vaccine efficacy are available outside western developed nations, although studies in the Gambia and South Africa have reported that multi-valent pneumococcal vaccines are safe and effective.^4^,^5^ The World Health Organization has recommended the inclusion of pneumococcal conjugate vaccine in national childhood immunization programs, particularly in countries where mortality rates among children less than five years of age are > 50/1,000 live births or where > 50,000 children die annually.^1^

Major initiatives are currently underway to introduce PCV-7 and to evaluate the efficacy of vaccination throughout the developing world.^6^–^8^ Serotypes included in PCV-7 (4, 6B, 9V, 14, 18C, 19F, and 23F) are reported to cover 60–90% of serotypes associated with IPD in young children in the developed world, although the prevalence of serotypes show geographic variability and coverage is suggested to be lower in many developing countries, such as those in Asia (45%).^9^,^10^ Other pneumococcal vaccines recently available in several countries include a 10-valent vaccine (PCV-7 serotypes plus serotypes 1, 5 and 7F; Synflorix™; GlaxoSmithKline, Brentford, United Kingdom), and a 13-valent vaccine (PCV-10 serotypes plus serotypes 3, 6A and 19A, Prevnar 13®; Wyeth Lederle Vaccines).

The incidence of IPD, circulating serotypes, and predicted vaccine efficacy in resource-poor Asia are poorly defined. Only a single, large-scale vaccine efficacy study has been conducted in the Philippines.^8^ In Laos, mortality in children less than five years of age was 70/1,000 in 2007. Immunization rates during the same year were low as reflected by a measles vaccination rate of 40%,^11^ and pneumococcal vaccination is not currently a component of the national program. Laos is one of several developing countries in Southeast Asia that has almost no or limited data on the burden of pneumococcal disease. A large study of the causes of community-acquired bacteremia at Mahosot Hospital, Vientiane (the capital of Laos), which included 4,512 blood culture pairs from adults and children during 2000–2004, provided the first data on *S. pneumoniae* for this country.^12^ The frequency of detected *S. pneumoniae* bacteremia was surprisingly low in children (2.1% of blood culture positive children) and adults (1.2% of positive adults). The serotypes responsible and outcome from invasive pneumococcal disease in Laos have not been reported.

We describe the burden of IPD among patients who provided blood and/or cerebrospinal fluid (CSF) for culture after admission to Mahosot Hospital over a period of six years and the serotypes of infecting pneumococci. The addition of a real-time polymerase chain reaction (PCR) for detection of *S. pneumoniae* in CSF led to a doubling in numbers of pneumococcal meningitis detected. To provide a more complete picture of circulating serotypes, we developed a real-time PCR serotyping method that encompassed the serotypes present in the 13-valent vaccine.

## Materials and Methods

### Study site.

A prospective study was conducted during January 2003–April 2009 at Mahosot Hospital, Vientiane, a 365-bed hospital with approximately 1,200 admissions per month. The study of IPD reported was part of a larger evaluation of all-cause sepsis and/or meningitis conducted over the same period. Ethical approval was granted by the Ethical Review Committee of the Faculty of Medical Sciences, National University of Laos and the University of Oxford Tropical Ethics Research Committee.

### Patient recruitment, sampling, and case definitions.

Patients were recruited after giving informed verbal consent for blood cultures (written informed consent from 2007) if community-acquired septicemia was suspected, and after informed written consent for lumbar puncture removal of CSF (parents or guardians were asked to determine consent for children) if meningitis was suspected. Recruitment was at the discretion of the responsible physician. History and clinical examination were recorded on a standard form. Two blood cultures were taken from each patient, as described.^12^ The volume of blood taken for culture was 5 mL/bottle for adults (≥ 15 years of age), 2 mL/bottle for children > 1 year of age, and 1 mL/bottle for infants. The blood culture bottles used varied according to patient age: bottles containing 50 mL of broth were used for adults, 20 mL for children, and 10 mL for infants. Immediately after lumbar puncture, 2–3 drops of CSF were placed onto a goat blood agar plate and a chocolate agar plate.

A case of IPD was defined as a patient who had a positive culture for *S. pneumoniae* in blood and/or CSF, and/or had a positive result for *S. pneumoniae* in CSF by a real-time PCR assay specific for *lytA*.^13^ Meningitis was defined as detection of *S. pneumoniae* in the CSF or in blood from a patient with a clinical syndrome consistent with meningitis.^1^ Patients with *S. pneumoniae* isolated from blood who had CSF that was negative for *S. pneumoniae* or did not have CSF taken and had no evidence for pneumococcal meningitis were categorized as having bacteremia. Death was recorded up to the time of hospital discharge.

### Microbiologic culture and bacterial identification.

Blood culture bottles were processed as described.^12^ The CSF samples and plates were transported immediately from the ward to the diagnostic laboratory. A cell count was performed on CSF by using a standard method.^14^ A total of 500 μL of CSF was centrifuged at 13,000 rpm for 5 minutes, the supernatant removed, and the material examined after Gram staining. The remaining pellet was divided between a goat blood agar plate, a chocolate agar plate, and tryptone soya broth, which was subcultured onto blood agar and chocolate agar after incubation at 37°C in an atmosphere of 10% CO_2_ for 24 hours. All agar plates were incubated at 37°C in an atmosphere of 10% CO_2_ for 72 hours and visually inspected daily. *Streptococcus pneumoniae* was identified by using standard microbiologic methods, which included colony morphology, Gram stain, optochin susceptibility, and bile solubility. All isolates were stored at –80°C in Protect™ Bacterial Preservation Cryovials (Fisher Scientific, UK). The serotypes of clinical *S. pneumoniae* isolates cultured at Mahosot Hospital were determined by using the Quellung reaction,^15^ which was performed at the John Radcliffe Hospital, Oxford, United Kingdom.

### Antimicrobial drug susceptibility testing.

Susceptibility to penicillin (minimum inhibitory concentration) was determined by using the Etest (AB Biodisk, Solna, Sweden), as described by the Clinical and Laboratory Standards Institute.^16^ The revised penicillin breakpoints (for intravenous administration) introduced in 2008 were used to define susceptibility of isolates associated with infections other than meningitis (susceptible, ≤ 2 μg/mL; intermediate, 4 μg/mL; and resistant, ≥ 8 μg/mL) and for isolates associated with meningitis (susceptible, ≤ 0.06 μg/mL; intermediate, none; and resistant, ≥ 0.12 μg/mL).^17^ Susceptibility to ceftriaxone, chloramphenicol, ofloxacin, and trimethoprim/sulfamethoxazole were also defined by Etests according to Clinical and Laboratory Standards Institute breakpoints.^16^ Susceptibility to erythromycin was determined by using the disk diffusion method,^16^ with intermediate and resistant isolates being grouped as nonsusceptible. If blood and CSF cultures were positive for *S. pneumoniae*, results for the isolate from CSF were presented.

### Laboratory isolates.

Three isolate collections were used during the development and validation of the real-time PCR serotyping assay. These were 1) a panel of reference *S. pneumoniae* isolates of known serotype (Table 1); 2) a collection of 121 additional *S. pneumoniae* isolates representing 13 vaccine serotypes (1, 3, 4, 5, 6A, 6B, 7F, 9V, 14, 18C, 19A, 19F, and 23F) and 35 isolates representing an additional 8 serotypes, which are either relatively common or belong to the same serogroup as found in PCV-13 (7B, 7C, 8, 9N, 19C, 23A, 23B, and 24A); and 3) a collection of non-pneumococcal isolates, with 1 isolate of each of the following species: *Enterococcus faecalis*, *E. faecium*, *Streptococcus bovis*, *S. mitis*, *Listeria monocytogenes*, *Haemophilus influenzae*, *H. parainfluenzae*, *Enterobacter cloacae*, *Shigella sonnei*, *Citrobacter freundii*, *Klebsiella pneumoniae*, and *Proteus mirabilis* (all obtained from the United Kingdom National External Quality Assessment Service scheme), *Staphylococcus aureus* ATCC 25923, *S. epidermidis* NCTC 11047, *S. viridans* NCTC 10712, *Neisseria meningitidis* NCTC 10025, *Esherichia coli* ATCC 25922, and *Pseudomonas aeruginosa* ATCC 27853. Additional species were included to represent pathogens that may cause central nervous system infection in Southeast Asia. These species were one clinical isolate each of *Streptococcus suis* and *Leptospira interrogans* serovar Autumnalis, and the reference strains *Orientia tsutsugamushi* strain Gilliam and *Rickettsia typhi* strain Wilmington (kindly provided by the Australian Rickettsial Reference Laboratory, Geelong, Victoria, Australia).

### DNA extraction from bacteria and CSF.

DNA was extracted from bacterial isolates by using the High Pure PCR Template Preparation Kit (Roche, Welwyn Garden City, UK) according to the manufacturer's protocol and the addition of mutanolysin (5 μL at a concentration of 10 mg/mL), lysostaphin (5 μL at a concentration of 10 mg/mL), and lysozyme (5 μL at a concentration of 10 mg/mL) (Sigma, St. Louis, MO) as appropriate at the cell lysis stage to promote lysis of *S. pneumoniae*, *S. aureus*, and coagulase-negative staphylococci. DNA was suspended in a final volume of 200 μL of buffer. DNA extraction from 200 μL of CSF was performed by using the QIAGEN DNA Mini protocol (Qiagen, Valencia, CA),^18^ with the modification that lysozyme (5 μL at a concentration of 10 mg/mL) and mutanolysin (5 μL at a concentration of 10 mg/mL) (Sigma) were added during a 30-minute lysis step at 37°C. DNA was eluted in 80 μL of Qiagen elution buffer.

### Real-time PCR to detect *S. pneumoniae* in CSF.

All CSF samples were tested for *S. pneumoniae* by using a real-time PCR specific for *lytA*, primers and probe described,^13^ and conditions described below.

### Development of molecular capsular typing by using real-time PCR.

#### Primer and probe design and multiplex PCR scheme.

Twelve primer pairs and fluorescent dye-labeled probes were designed to target the serotypes present in the 13-valent vaccine (serotypes 1, 3, 4, 5, 6A/B, 7F, 9V, 14, 18C, 19A, 19F, and 23F) (a single serotype 6 assay would be predicted to amplify 6A/B/C). The relevant *cps* gene sequences for each serotype were downloaded from the National Center for Biotechnology Information (Bethesda, MD) website (http://www.ncbi.nlm.nih.gov/) and aligned. Differences between serotypes within the same serogroup were highlighted and primers and locked nucleic acid probes for use in Taqman PCRs were selected and synthesized by Sigma Aldrich (Dorset, United Kingdom). Serotype-specific genes selected from the *cps* locus were *wchD* (serotype 1), *wchE* (serotype 3), *wzy* (serotype 4), *whaC-whaD* (serotype 5), *wzy-wzx* (6A/B/C), *aliB* (7A/F), *cps9vJ* (9A/V), *wzy* (14), *gct* (18B/C), *mnaA* (19A), *wzy* (19F), and *wzy-wchV* (23F).

Each primer pair and probe were compared with all *cps* sequences using the basic local alignment search tool (BLAST) website (http://blast.ncbi.nlm.nih.gov/Blast.cgi) and BLAST search on the Sanger website (http://www.sanger.ac.uk/Projects/S_pneumoniae/CPS/) to determine specificity. The probes were labeled at the 5′ end with either 6-carboxyfluorescein (FAM; serotypes 3, 7A/F and 19F), hexachloro-6-6-carboxyfluorescein (HEX; serotypes 1, 6A/B/C, 18B/C and 19A), cyanine (Cy5; serotypes 4, 9A/V, and 23F), or 6-carboxy-X-rhodamine (ROX; serotype 5 and 14). Black hole quencher (BHQ; Sigma) was attached at the 3′ end of each probe (either BHQ1 or 2, depending on the probe dye). Primer and probe sequences are shown in Table 2. Primers and probes were grouped into three multiplex reactions and one single target reaction. Multiplex 1 was designed to amplify serotypes 1, 3, 4, and 5, multiplex 2 to amplify 6A/B/C, 7A/F, 9A/V, and 14, multiplex 3 to amplify 18B/C, 19F, and 23F, and the single target reaction for serotype 19A.

#### Real-time PCRs.

All PCRs were optimized for the Corbett Rotor-Gene 6000 series (Corbett Life Science, Sydney, New South Wales, Australia) in a 25-μL reaction containing 1× PCR buffer, 5.5 mM MgCl_2_, 200 μM of each dNTP, variable primer and probe concentration (see below), 1 unit of AmpliTaq Gold DNA polymerase, and 3 μL of DNA extract from CSF samples or 2 μL of extracted bacterial DNA. After optimization, the primer and probe concentrations used were as follows: each primer, *lytA*, 300 nM; capsular multiplex reactions 1 and 2, 240 nM; multiplex reaction 3 and single target reaction, 300 nM; each probe: *lytA*, 100 nM; multiplex 1 and 2, 40 nM of FAM-labeled probes (serotypes 3 and 7A/F) and 80 nM each of probes labeled with other dyes (serotypes 1, 4, 5, 6A/B/C, 9A/V, and 14); multiplex 3, 50 nM of FAM-labeled probe (serotype 19F) and 100 nM each of probes labeled with other dyes (serotypes 18B/C and 23F); and single reaction, 100 nM of probe (serotype 19A). DNA was amplified by using 45 cycles at 95°C for 15 seconds and 60°C for 60 seconds.^19^ Amplification data were analyzed by using Rotorgene 6000 software version 1.7 (Corbett Life Science). The relevant positive control (2 μL of genomic DNA) and negative control (reaction mixture minus template) were included in each run. Positive samples were defined as those with a cycle threshold (C_T_) < 40. The lower limit of detection was determined by using serial 10-fold dilutions of purified DNA from known pneumococcal serotypes.

## Results

### Detection of *S. pneumoniae* in blood and CSF.

Blood cultures were obtained from 10,799 patients during January 2003–April 2009 for the investigation of suspected bacterial sepsis, of which 23 (0.21%) were culture positive for *S. pneumoniae*. A total of 353 patients had a CSF sample taken for investigation of suspected meningitis, of which 19 (5.4%) were defined as having pneumococcal meningitis on the basis of culture and positive *lytA* PCR results (n = 9), or positive *lytA* PCR results alone (n = 10). Seven patients were positive for *S. pneumoniae* in blood culture and CSF samples, 12 patients had a positive CSF result and a negative blood culture, and 16 patients had a positive blood culture alone. Therefore, we describe 35 patients with IPD. Features in patients with positive blood cultures alone were no known focus of infection (n = 8), pneumonia (n = 7), and clinical meningitis with no CSF sample taken (n = 1).

The median age of the 35 patients with IPD was 17 years (range = 1 month to 82 years, interquartile range = 1.5–34.8 years); 11 (31%) patients were less than five years of age. The crude in-hospital mortality rate was 31% (11 of 35). Of those who died, five patients had bacteremic meningitis, two had meningitis, and four had bacteremia alone. The median age in patients who died was 20 years (range < 1–63 years).

### Antimicrobial drug susceptibility.

Susceptibility testing was completed for isolates from 23 of the 35 patients. This testing could not be completed for 12 patients because the diagnosis was based on a positive *lytA* PCR result only for CSF (n = 10) or because isolates could not be recovered from frozen stocks (n = 2). Two isolates associated with meningitis were not susceptible to penicillin (minimum inhibitory concentrations = 0.39 μg/mL and 0.125 μg/mL, respectively). All 23 *S. pneumoniae* were susceptible to ceftriaxone and ofloxacin. Resistance patterns to other antimicrobial agents were erythromycin, 3 isolates (13%); chloramphenicol, 3 isolates (13%); and trimethoprim-sulfamethoxazole 14 isolates (61%). Seven isolates (30%) were fully susceptible to all antimicrobial drugs tested, six isolates (26%) were resistant to one antibiotic, six isolates (26%) were resistant to two antibiotics, and four isolates (17%) were resistant to ≥ 3 of the antimicrobial agents tested.

### Sensitivity and specificity of real-time PCR serotype assay.

The lower limit of detection was defined for the 12 serotype-specific PCR assays by using serial 10-fold dilutions of purified DNA extracted from the relevant control strains for each pneumococcal serotype. The lower limit of detection in the multiplex reactions ranged from 0.35 to 26.4 genomic equivalents per reaction. Verification of assay specificity was performed by using genomic DNA from a range of other bacterial species. No amplification products were detected in the three multiplex or one single target reactions for any of the non-pneumococcal species tested, which indicated 100% assay specificity for *S. pneumoniae*. Specificity of the PCR primers for specific serotypes was assessed by using the panel of reference isolates of known serotypes (Table 1). Primers were shown to be serotype specific with no cross-reactions between serotypes.

### Validation and application of pneumococcal serotyping by using real-time PCR.

A blinded evaluation was performed in which serotype determined by the Quellung reaction and the PCR were compared for 121 *S. pneumoniae* isolates with serotypes represented in the PCV-13 vaccine (Table 3). There was 100% concordance between these two assays. An additional 35 isolates representing other serotypes as defined by the Quellung reaction that are not in the PCV-13 vaccine but that have been reported to cause IPD worldwide or are closely related to the vaccine serotype were negative by the PCR.

The 10 CSF samples that were culture negative but positive by *lytA* PCR were evaluated by real-time PCR to determine the serotype of the infecting isolate. The serotypes were type 1 (n = 2), 5 (n = 2), 6A/B/C (n = 1), and undetermined (no product) (n = 5). These data enabled us to define the serotype of 28 of 35 patients with IPD (not including the 5 unknowns and 2 isolates that could not be recovered from frozen stocks). Summary data for pneumococcal serotypes as defined by Quellung and PCR or PCR alone are shown in Table 4. The most frequent serotype defined was 1 (n = 6), followed by serotypes 5, 6A/B/C, 14, and 23F.

Overall, 25 (76%) of 33 patients were infected with a strain that was represented in the PCV-13 vaccine, with coverage of 73% (24 of 33) for the PCV-10 vaccine and 39% (13 of 33) for the PCV-7 vaccine. Serotype 6 strains (6A, 6B, and 6C) were considered as a single entity for this calculation because they were indistinguishable by PCR. If one considers only those cases in children less than five years of age (n = 11), coverage for PCV-13, PCV-10, and PCV-7 was 73%, 73%, and 45%, respectively. Only seven cases were in children less than two years of age, four (54%) of whom had IPD caused by a strain represented in the PCV-7 vaccine.

## Discussion

This is the first study of IPD in Laos and presents the first data on *S. pneumoniae* serotypes of invasive isolates in this setting. We found that *S. pneumoniae* was rarely identified as a cause of bacterial infection in comparison with studies conducted in Europe and North America.^9^,^10^ Possible explanations for this finding are either that the rate of pneumococcal carriage (the forerunner to disease) is low in our setting, or that IPD was more common than these culture and/or PCR results suggest, with false-negative culture and PCR results leading to an underestimation of the true incidence of IPD. Because the rate of pneumococcal carriage has not been reported for any age group in Laos, the first possibility remains to be tested, although children 1–16 years of age living in nearby Vietnam have been reported to have a carriage rate of 44%.^20^ A study conducted in Thailand reported an annual incidence rate for hospitalized pneumococcal bacteremia of 3.7 and 7.6 cases per 100,000 persons for two rural provinces, respectively.^21^ The possibility that our study underestimates the true incidence of IPD is highly plausible, especially because of the high frequency of consumption of antimicrobial drugs prior to culture in our patient population. A wide range of over-the-counter antibiotics are available in Laos, and self-medication is common during a febrile illness. This finding is supported by recent evidence that 56.8% of patients admitted in Mahosot Hospital whose investigations included a lumbar puncture had antimicrobial drug activity detected in their urine (Khennavong M, unpublished data). The number of CSF samples that were positive for *S. pneumoniae* doubled when examined by using PCR, and the application of molecular methods to blood samples could lead to a further increase in detection of IPD.

Although the number of clinical isolates available for testing in this study was low, we found that serotypes 1, 5, 6A/B, 14, and 23F were most commonly identified. A study of pneumococcal carriage in children in Vietnam reported that serotypes 23F, 19F, 6B, and 14 predominated.^22^ In Thailand, studies of *S. pneumoniae* isolated from patients admitted to hospitals have demonstrated some variation in the predominant serotypes, with 23F, 19F, and 6B predominating in a study conducted in northern regions,^23^ serotypes 6, 23, 19, 3, and 11 predominating in Bangkok,^24^ and serotypes 14, 6B, 3, and 19A predominating in the northeastern regions.^21^ A study (Asian Network for Surveillance of Resistant Pathogens) of 996 isolates from 11 countries in Asia demonstrated that serotypes 23F and 19F predominated, followed by serotypes 6 and 14,^25^ and the same four serotypes were reported to predominate in Colombo, Sri Lanka.^26^ The five most common serotypes causing invasive pneumococcal infection in China were reported to be 19F, 14, 19A, 6B, and 23F.^27^ Thus, our finding that serotype 1 predominated is different from data for several countries in the region, although it is similar to a recent study conducted in Nepal where serotype 1 predominated, followed by 5, 2, and 7F.^28^ Serotype 1 is often associated with outbreaks, but patients in this study infected by serotype 1 isolates were not related in time or place.

The small number of isolates we were able to examine will necessarily mean that our data have wide error margins, and further studies are required with larger numbers of isolates. Our finding that PCV-7 vaccine coverage would be predicted to be low in Vientiane is a potentially important observation for Laos, and further studies are urgently required to confirm this finding and determine whether this is the case elsewhere in the country. This finding of low vaccine coverage by PCV-7 is not unique in our region; a rate of 51% has been reported in a study in nearby northeastern Thailand.^21^ The estimated vaccine coverage for Thailand is similar when isolates associated with carriage are considered.^29^,^30^

The use of PCR more than doubled the estimated burden of *S. pneumoniae* associated with meningitis compared with culture alone. Our real-time PCR identified those serotypes in the PCV-13 vaccine and gave results that were concordant with those of the Quellung reaction, and without cross-reactivity with other potential pathogens causing meningitis. This assay could be used to define prevalent serotypes prior to vaccination programs in settings where patients commonly use antimicrobial drugs prior to hospital presentation. Our results echo the findings from a study of pneumococcal disease in children in Vietnam, in which culture was supported by antigen detection by using an immunochromatographic test and conventional PCR.^31^ Culture only confirmed one case of *S. pneumoniae*, with an additional 16 children identified as having pneumococcal disease on the basis of antigen detection (n = 12) or PCR (n = 4).^31^ Previous studies have used conventional PCR to determine serotype by using *cps* genes as targets.^32^–^34^ However, these studies used numerous multiplexes in a single reaction or a number of different multiplex reactions and steps to deduce the serotype. Although a real-time PCR approach uses multiple reactions, the sensitivity is higher than that of conventional PCR. The increased sensitivity of real-time PCR could lead to introduction of the technique into the clinical setting, especially for samples in which bacterial load is low.

A limitation of our PCR assay is that some highly related serotypes that can be distinguished by the Quellung reaction cannot be distinguished by the PCR. For example, 6A, 6B, and 6C serotypes, although not always easy to distinguish by the Quellung reaction,^35^ were grouped in our PCR and require a detailed scheme to distinguish between them by using a molecular approach.^36^,^37^ Both 6A and 6B are found in the 13-valent pneumococcal vaccine, and an additional probe that can distinguish 6C may be advantageous because cross-reactivity has been reported to occur between 6A and 6B, but not with 6C.^38^ Similar to other assays,^32^,^33^ our assay also failed to distinguish between serotypes 7A and 7F, 9A and 9V, and 18B an 18C. Those serotypes (7A, 9A, and 18B) not in the PCV-7 vaccine are reportedly rare worldwide. There may be some cross-protection between serogroups found in the vaccine, although this has yet to be determined for many of the serotypes.^39^ Our strain collection did not contain serotypes 7A or 9A as defined by Quellung serotyping.

Because the incidence of IPD in this study was low and coverage using the PCV-7 vaccine would be predicted to be relatively low, it is a matter for debate as to whether a pneumococcal vaccine should be a high priority public health intervention in Laos until such times as there is evidence to support this suggestion. Further studies are urgently needed to determine whether these findings are reproducible elsewhere in Laos, to determine whether this represents a true picture of the burden of IPD or is an underestimate, and to define the most appropriate vaccine for the prevention of pneumococcal disease in this setting.

## Figures and Tables

**Table 1:** *Streptococcus pneumoniae* reference isolates used in the study, Laos

Source	Strain no.	*S. pneumoniae* serotype
Health Protection Agency Culture Collection, London, United Kingdom	NCTC 7465	1
	NCTC 7978	3
	NCTC 11886	4
	NCTC 11887	5
	NCTC 11888	6B
	NCTC 11890	7A
	NCTC 11889	7F
	NCTC 11894	9A
	NCTC 11895	9L
	NCTC 11896	9N
	NCTC 11897	9V
	NCTC 11902	14
	NCTC 11905	18C
	NCTC 11907	19A
	NCTC 12977	19F
	NCTC 11910	23F
Statens Serum Institut, Copenahgen, Denmark	SSISP 18A/2	18A
	SSISO 18B/2	18B
	SSISP 18F/1	18F

**Table 2:** Primers and probes used in the study, Laos*

Serotype	Accession no.	Primer/probe	Sequence (5′→3′)	Primer position at *cps* locus	Product size, basepairs
1	CR826497.1	For	CTATAGAAGGTCTACATCAGGTTC	8626–8784	159
		Rev	TTTCTGTCAGATACGGCTTAC		
		Probe	HEX-TCT+TCA+ATG+CGT+AGT+CTGC-BHQ1		
3	Z47210.1	For	ATGTTATTACACTCCTGTTCCTG	7282–7413	132
		Rev	TCTAGGCGTCCATACTGTATC		
		Probe	FAM-AGA+ACT+GTA+ATA+TCA+CTCTGCGA-BHQ1		
4	AF316639.1	For	TATTTCTAGGGTAATAACTGATTCTAAAAC	9990–10099	110
		Rev	CTCCTAAATCATCTATTATTCCTGAAC		
		Probe	Cy5-CTG+CCT+CTG+AAT+ATG+CTGAAT-BHQ2		
5	CR931637.1	For	TCCGAACGAAGATATTTGGTG	9143–9252	110
		Rev	ATATAGAATTCCCCTCATGAACAC		
		Probe	ROX-ACC+ACA+ACA+TCC+TCA+ATCAAC-BHQ2		
6A/B/C	CR931638.1	For	TATTATTCTTTAGGGAATGTGTATACTG	10290–10391	102
		Rev	ATATAACCACGCTGTAAAACTC		
		Probe	HEX-CAA+TAC+CAA+TTA+CAC+CAAAGTCT-BHQ1		
7A/F	CR931643.1	For	CCTTATAAATTTTGTGACTATAGACCTG	9799–9902	104
		Rev	CCTAGTAAGACATCTGTGTCAC		
		Probe	FAM-AAC+CCC+AGT+AAT+CAT+AACCC-BHQ1		
9A/V	CR931648.1	For	GTTAGTTGCTTCTTACAGGAAATAC	10014–11411	115
		Rev	AAATTCATATTCCCACTCATTGTATG		
		Probe	Cy5-ACT+TCC+ATC+AGT+AAG+CAGTTT-BHQ2		
14	CR931662.1	For	TCTATATACAAAGAGGCTCCAATG	7492–7592	101
		Rev	ACCTGTATATCTTACACCATAACTAG		
		Probe	ROX-AAA+TCC+GTC+CCA+GTC+TAAC-BHQ2		
18B/C	CR931673.1	For	TCGATTTAGTAATCCCTGAAAC	15812–15922	111
		Rev	GATAATCAAATTTACCTTTCCAATC		
		Probe	HEX-TCA+GAT+GTT+AAA+GACTACC-BHQ1		
19A	CR931675.1	For	AGAGGATTATACACACACTCATTTAG	12650–12730	81
		Rev	AGATTTTCGCGTCTATGAGC		
		Probe	HEX-CGC+TAA+CAA+TCG+TCT+CATCTT-BHQ1		
19F	CR931678.1	For	TCGGACACTAGGAGTTACTG	11848–11955	108
		Rev	AAAGCACCTACAGCAAAGAC		
		Probe	FAM-ACA+TAC+ATA+CCA+ACT+AGA+CCAA-BHQ1		
23F	CR931685.1	For	GAACGGTAGAGATGCCTTTAC	9273–9366	94
		Rev	GAAGATATAAACTTAAACAGCACTATAATG		
		Probe	Cy5-CAA+CTA+ACC+CAA+CAT+AAC+CATTT-BHQ2		

* For = forward; Rev = reverse.

**Table 3:** Concordance between serotype as defined by the Quellung reaction and real-time PCR assay for 121 laboratory isolates of *Streptococcus pneumoniae*, Laos*

Serotype by Quellung reaction	Serotype by real-time PCR	No. isolates tested
1	1	7
3	3	9
4	4	8
5	5	8
6A/B/C†	6A/B/C	23
7A/F†	7A/F	12
9A/V†	9A/V	13
14	14	9
18B/C†	18B/C	6
19A	19A	9
19F	19F	8
23F	23F	9
Total		121

* PCR = polymerase chain reaction. Results are shown for serotypes present in the 13-valent polysaccharide-protein conjugate vaccine. Concordance was 100% for all serotypes tested.

† These results include the following Quellung results: 6A, n = 9; 6B, n = 9; 6C, n = 5; 7A, n = 3; 7F, n = 9; 9A, n = 4, 9V, n = 9; 18B, n = 1; and 18C, n = 5. An additional 35 strains representing additional Quellung-defined serotypes not present in this vaccine (7B, n = 3; 7C, n = 6; 8, n = 2; 9N, n = 8; 23A, n = 9; 23B, n = 6; and 24A, n = 1) were tested by real-time PCR and gave negative results.

**Table 4:** Serotypes of 33 pneumococcal isolates associated with invasive pneumococcal disease in Laos*

Serotype†	Quellung reaction and PCR (n = 23)	PCR positive alone (n = 10)	Total (n = 33)	Children < 5 years of age	Associated with meningitis/< 5 years of age
1	4	2	6	1	3/1
4	2	0	2	0	1/0
5	2	2	4	2	2/2
6A/B/C	2	1	3	0	2/0
7F	1	0	1	0	0
9V	1	0	1	0	0
14	3	0	3	3	2/2
18C	1	0	1	0	1/0
19A	1	0	1	0	1/0
23F	3	0	3	2	1/0
15C	1‡	0	1	1	0
23B	1‡	0	1	0	1/0
38	1‡	0	1	0	0
Unknown	0	5	5	2	5/2

* PCR = polymerase chain reaction.

† No serotype 3 or 19F isolates were found in our collection.

‡ Determined by Quellung alone as primers for serotypes 15C, 23B, and 38 are not included in real-time PCR assay results.
